# Cats as a Risk for Transmission of Antimicrobial Drug-resistant *Salmonella*

**DOI:** 10.3201/eid1012.040904

**Published:** 2004-12

**Authors:** Filip Van Immerseel, Frank Pasmans, Jeroen De Buck, Ivan Rychlik, Helena Hradecka, Jean-Marc Collard, Christa Wildemauwe, Marc Heyndrickx, Richard Ducatelle, Freddy Haesebrouck

**Affiliations:** * Ghent University, Merelbeke, Belgium;; † Veterinary Research Institute, Brno, Czech Republic;; ‡ Scientific Institute of Public Health, Brussels, Belgium;; § Pasteur Institute of Brussels, Brussels, Belgium;; ¶ Center for Agricultural Research, Melle, Belgium

**Keywords:** zoonoses, *Salmonella* Infections, cats, disease reservoirs, research

## Abstract

To determine whether cats were a risk for transmission of *Salmonella* to humans, we evaluated the excretion of *Salmonella* by pet cats. Rectal-swab specimens were taken from 278 healthy house cats, from 58 cats that died of disease, and from 35 group-housed cats. Group-housed cats were kept in one room with three cat trays and a common water and feed tray. Eighteen (51.4%) of 35 group-housed cats, 5 (8.6%) of 58 diseased cats (5/58), and 1 (0.36%) of 278 healthy house cats excreted *Salmonella*. *Salmonella* isolates were of serotypes Typhimurium, Enteritidis, Bovismorbificans and 4:i:-. Acquired antimicrobial resistance was found in serotype Typhimurium (resistance to ampicillin, chloramphenicol, and tetracycline; to ampicillin; and to chloramphenicol) and 4:i:- strains (resistance to ampicillin, chloramphenicol, sulfonamides, trimethoprim, and sulfamethoxazole/trimethoprim). Cats that excrete *Salmonella* can pose a public health hazard to people who are highly susceptible to *Salmonella*, such as children, the elderly, and immunocompromised persons.

## Abstract 2

Cats can shed antimicrobial drug−resistant *Salmonella* serotypes in the environment.

*Salmonella* infections are still a leading cause of human foodborne infections in the world (*1**,**2*). These infections primarily originate from eating contaminated food, especially chicken eggs and egg products, and also meat products from pigs and chickens (*3**,**4*). Considering the high frequency of food contamination and the emergence of multidrug-resistant *Salmonella* strains, control of *Salmonella* in food-producing animals has become a worldwide challenge. Other environmental sources can lead to accidental human infections with *Salmonella* as well. The role of pet animals as a source of *Salmonella* has not been fully investigated, but severe human infections originating from reptiles, especially pet turtles, have been reported (*5*).

Cats and dogs are the most widely kept pet animals, yet the incidence of *Salmonella* in these animals is largely unknown, and the risk that these animals pose for transmission of *Salmonella* to humans is unclear. In particular, cats that can freely roam outside, and are therefore able to scavenge or hunt food of unknown quality, are potential candidates for *Salmonella* carriage. Most reports concerning *Salmonella* and cats are case studies of clinical salmonellosis, which resulted in septicemia and death (*6**,**7*). Subclinical infections and carrier animals, however, are much more important with respect to transmission to humans. In this study, rectal swabs from cats of different origin (house cats, group-housed cats, diseased cats) were cultured for *Salmonella*. The serotype and phage type of the *Salmonella* isolates were determined, and the isolates were characterized with respect to their antimicrobial drug–resistance pattern and interaction with human intestinal epithelial cells.

## Methods

### Collection of Fecal Samples

A total of 278 rectal swab samples from house cats of different age, sex, and breed were taken between July and November 2003. All house cats came from different owners. The animals came from all over the Dutch-speaking part of Belgium, i.e., north of Brussels. Rectal swab specimens were also taken from 58 cats that were submitted for autopsy to the Faculty of Veterinary Medicine, Ghent University. The latter died or were euthanized because of incurable disease. All cats came from different owners, except three cats that had feline immunodeficiency virus (FIV), which came from one owner. Finally, rectal samples of 35 kittens (all <4 months of age) were taken at a facility where the animals were group-housed, waiting to be adopted. These animals came from 16 different owners.

### Bacteriologic Analysis

Bacteriologic analysis was performed by enrichment of the rectal swabs. The samples were first pre-enriched in buffered peptone water (BPW) (Oxoid, Basingstoke, Hampshire, UK) overnight at 37°C, after which 1 mL of this suspension was added to 9 mL of tetrathionate brilliant green broth (Oxoid) (enrichment). After incubation overnight at 37°C, a drop of this suspension was spread on brilliant green agar (BGA) (Oxoid). Both the serotype and phage type of positive isolates were determined.

### Antimicrobial Susceptibility Testing

Resistance to antimicrobial agents was tested by using the disk diffusion assay on Mueller-Hinton agar with commercial antimicrobial susceptibility disks (Oxoid) according to the international standards of the National Council for Clinical Laboratory Standards (NCCLS) (*8*). The following antimicrobial agents were tested: ampicillin (A, 10 μg), chloramphenicol (C, 30 μg), streptomycin (S, 10 μg), sulfonamide (Su, 300 μg), tetracycline (T, 30 μg), ciprofloxacin (Cip, 5 μg), kanamycin (K, 30 μg), gentamicin (Gn, 10 μg), sulfamethoxazole-trimethoprim (Sxt, 25 μg), cefotaxime (Cxt, 30 μg), nalidixic acid (Na, 30 μg), and amoxicillin-clavulanic acid (Amc, 30 μg). *Salmonella enterica* serovar Typhimurium 8420 (resistance type ACSSuT), 6237 (sensitive), 3520 (resistance type T), 2200 (resistance type ASSuT), and 5833 (sensitive) isolates from human patients in Belgium were used as control strains in antimicrobial susceptibility testing.

### Polymerase Chain Reaction (PCR)

For PCR, a loop of bacterial culture was resuspended in 50 μL of water, and DNA was released from bacterial cells by boiling for 20 min. After the mixture was spun for 1 min in a microfuge at 14,000 x *g*, 2 μL of the supernatant was taken as a template DNA for PCR. PCR was carried out in 20-μL volumes by using PCR Master Mix from Qiagen (Hilden, Germany), according to the manufacturer's instructions. All the resistant strains were tested for the presence of the genes typical for particular resistance. The genes determined and primers used are listed in Table 1. Cycling consisted of 50-s incubations at 92°C, 55°C, and 72°C, which were repeated 25 times. After PCR, amplification products were detected by electrophoresis in 2% agarose gel, stained with ethidium bromide, and visualized under UV light. Antimicrobial drug-sensitive strain *S*. Typhimurium F98 was used as a negative control in all the amplifications. *S*. Typhimurium strains 8420, 6237, 3520, 2200, and 5833 were used as positive controls.

**Table 1:** List of primers used in the PCR reactions for detection of resistance genes

Resistance	Gene	Primer	Sequence (5´– 3´)	Size (bp)	Reference
Ampicillin	*blaPSE­1*	PSEF	TAG CCA TAT TAT GGA GCC TC	321	AF261825
		PSER	TTA ACT TTT CCT TGC TCA GC		
	*bla^TEM^*	TEMF	GCA CGA GTG GGT TAC ATC GA	310	9
		TEMR	GGT CCT CCG ATC GTT GTC AG		
	*blaoxa1*	oxa1F	AGC AGC GCC AGT GCA TCA	708	10
		oxa1R	ATT CGA CCC CAA GTT TCC		
Chloramphenicol	*floR*	floRF	GCG ATA TTC ATT ACT TTG GC	425	11
		floRR	TAG GAT GAA GGT GAG GAA TG		
	*Cat*	catF	CCT GCC ACT CAT CGC AGT	623	10
		catR	CCA CCG TTG ATA TAT CCC		
Streptomycin	*aadA1*	aad1For	CGA CTC AAC TAT CAG AGG TA	384	AY534545
		aad1Rev	CTT TTG TCA GCA AGA TAG CC		
	*aadA2*	aadA2F	CGG TGA CCA TCG AAA TTT CG	249	12
		aadA2R	CTA TAG CGC GGA GCG TCT CGC		
	*strA*	strAF	CCT ATC GGT TGA TCA ATG TC	250	11
		strAR	GAA GAG TTT TAG GGT CCA CC		
Tetracycline	*tetA*	tetAF	GCT ACA TCC TGC TTG CCT TC	210	13
		tetAR	CAT AGA TCG CCG TGA AGA GG		
	*tetB*	tetBF	TTG GTT AGG GGC AAG TTT TG	659	13
		tetBR	GTA ATG GGC CAA TAA CAC CG		
	*tetC*	tetCF	GCG GGA TAT CGT CCA TTC CG	207	14
		tetCR	GCG TAG AGG ATC CAC AGG ACG		
	*tetG*	tetGF	GCT CGG TGG TAT CTC TGC TC	468	13
		tetGR	AGC AAC AGA ATC GGG AAC AC		
Sulfonamide	*sul1*	sul1F	ATG GTG ACG GTG TTC GGC ATT CTG	841	15
		sul1R	GCT AGG CAT GAT CTA ACC CTC GG		
	*sul2*	sul2F	AGG GGG CAG ATG TGA TCG AC	249	11
		sul2R	GCA GAT GAT TTC GCC AAT TG		
Trimethoprim	*dfrA1*	dfrA1F	GTG AAA CTA TCA CTA ATG G	470	10
		dfrA1R	CCC TTT TGC CAG ATT TGG		
	*dfrA10*	dfrA10F	TTA ATT ACC AGA GCA TTC GG	374	AY049746
		dfrA10R	TAC ACA TCA GCA TGA ACA GG		
	*dfrA12*	dfrA12F	ACT CGG AAT CAG TAC GCA	463	10
		dfrA12R	GTG TAC GGA ATT ACA GCT		
Kanamycin	*aadD*	aadD*¬*F	ATA TTG GAT AAA TAT GGG GAT	161	12
		aadD*¬*R	TCC ACC TTC CAC TCA CCG GTT		
	*aphA/aph(3´)¬Id*	aphAaphIdF	ATG GGC GCC TAT CAC AAT TGG	257	12
		aphAaphIdR	TCG CCT CCA GCT CTT CGT AGA		
	*aphAI¬IAB*	aphAI*¬*IABF	AAA CGT CTT GCT CGA GGC	461	12
		aphAI*¬*IABR	CAA ACC GTT ATT CAT TCG TGA		
	*aph(3´)¬IIa*	KanAphF	GAG AAA GTA TCC ATC ATG GC	465	L19385
		KanAphR	GCT CAG AAG AAC TCG TCA AG		

All *Salmonella* strains were tested for the presence of the *SopB* gene. The primers were GATAGGAAAGATTGAGCACCTCTG and TACAGAGCTTCTATCACTCAGCTTC, and the PCR cycle consisted of 30 cycles of (30 s 95°C, 1 min 58°C, 1 min 72°C).

### Pulsed-Field Gel Electrophoresis (PFGE)

The bacteria were grown while being shaken overnight at 37°C in Luria-Bertani broth (LB). The *Xba*I PFGE patterns were determined for all 21 *S*. Typhimurium strains by using previously described PFGE methods (*16**,**17*) with some slight modifications. The patterns were grouped in a dendrogram with GelCompar II software (Applied Maths, St.-Martens-Latem, Belgium) by using the Dice coefficient and the unweighted pair group method with an arithmetic averages clustering algorithm.

### Invasion of the Human intestinal Epithelial Cell Line T84

The capacity of all cat *Salmonella* isolates and the human *S*. Typhimurium isolates 8420, 6237, 3520, 2200, and 5833 to invade human intestinal epithelial cells was determined. Cells of the human colon carcinoma cell line T84 were seeded in 96-well cell culture plates (Greiner, Frickenhausen, Germany) at a density of 5.10^5^ cells/mL culture medium (DMEM + 10% fetal calf serum + 2% L-glutamine, without antimicrobial drugs) and grown for 24 h. Bacteria were grown for 20 h in LB-medium, after which the suspension was diluted 1:50 in fresh LB-medium. After 4 h of incubation at 37°C, suspensions were centrifuged and resuspended in DMEM with 10% fetal calf serum (FCS). The number of colony-forming units (CFU)/mL was determined by plating 10-fold dilutions on BGA. The suspensions were stored overnight at 4°C. The next day, 10^6^ CFU in 200 μL were added to the T84 cell cultures, which were then centrifuged for 10 min at 1,500 rpm to make close contact between the bacteria and the colon cells. The plates were incubated for 1 h at 37°C and 5% CO_2_. The cells were then rinsed three times with Hanks' Balanced Salt Solution (HBSS, Life Technologies, Paisley, Scotland). Cell culture medium with gentamicin (50 μg/mL) was added, and plates were incubated for 1 h at 37°C and 5% CO_2_. Hereafter, the cells were rinsed three times with PBS and analyzed with 1% Triton X-100 (Sigma, St. Louis, MO) in distilled water. From this lysate, 10-fold dilution series were made. From each dilution, 6 x 20 μL was added to BGA, to determine the number of CFU *Salmonella* per mL. The assays were performed in triplicate. The percentage of intracellular bacteria, relative to the number of *Salmonella* bacteria, initially incubated with the cells, was calculated. The previously mentioned human isolates of *S*. Typhimurium were used for comparison between the cat isolates and human isolates. Statistical analysis was performed by analysis of variance methods using the SPSS 11.0 software.

## Results

### Characterization of *Salmonella* Isolates from Cats

**Figure 1:**
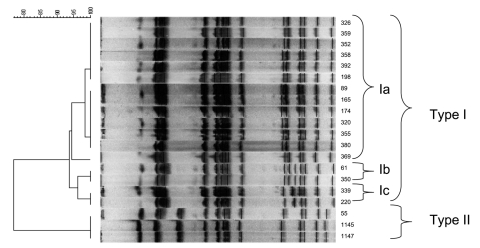
Dendrogram obtained by unweighted pair group method using arithmetic averages clustering of the pulsed-field gel electrophoresis–*Xba*I patterns of serovar Typhimurium strains isolated from cats on the basis of the Dice coefficient.

Of 278 healthy house cats, 1 *Salmonella* strain was isolated, an *S*. Enteritidis phage type 21 strain, sensitive to all tested antimicrobial drugs. Five strains were isolated from cats that died from or were euthanized because of incurable disease. Feline AIDS (caused by feline immunodeficiency virus [FIV]) was diagnosed in three cats, one died due to feline panleukopenia parvovirus infection, and one was poisoned. Three isolates were identified as being ampicillin-resistant *S*. Typhimurium phage type 193, harboring the *bla_TEM_* gene. They had the same pulsed-field gel electrophoresis (XbaI) pattern, indicating that the isolates were of clonal origin (Figure 1). The three cats came from the same owner. One isolate was an antimicrobial drug–sensitive *Salmonella* Bovismorbificans strain. One isolate was *Salmonella* 4:i:-, which was resistant to ampicillin, chloramphenicol, sulfonamides, tetracycline and sulfamethoxazole-trimethoprim (ACSuTSxt), harboring the *bla_TEM_*, *cat*, *sul2*, *tet*(*A*), and *dfrA1* antimicrobial drug–resistance genes. Eighteen strains were isolated from the group-housed cats. All of these were *S*.Typhimurium phage type 120/ad. Fourteen of these strains showed acquired resistance to ampicillin, chloramphenicol and tetracycline and harbored the *bla_TEM_*, *cat*, and *tet*(*A*) antimicrobial drug-resistance genes, while four isolates were resistant to chloramphenicol only and only harbored the *cat* gene (Table 2). Pulsed-field gel electrophoresis showed that the isolates from the group-housed cats were of the same *Xba*I PFGE type, and that three subtypes within this type were present, indicating a clonal origin (Figure 1). One subtype contained the 14 strains that were resistant to the three mentioned antimicrobial drugs. All *Salmonella* strains harbored the *SopB* gene.

**Table 2:** Characteristics of *Salmonella* isolates from cats^a^

Isolate no.	Source	Serotype	Phage type	PFGE pattern^b^	Resistance phenotype^b^	Resistance genotype
11	Diseased house cat	4:i:-	–	ND	ACSuTSxt	*bla_TEM_*, *cat*, *sul2*, *tet*(*A*), *dfrA1*
40	Diseased house cat	Bovismorbificans	–	ND	–	-
109	House cat	Enteritidis	21	ND	–	-
1145, 1147, 55	Diseased house cats	Typhimurium	193	II	A	*bla_TEM_*
89, 165, 174, 198, 320, 326, 352, 355, 358, 359, 369, 380, 390, 392	Group-housed cats	Typhimurium	120/ad	Ia	ACT	*bla_TEM_, cat, tet*(*A*)
161, 350	Group-housed cats	Typhimurium	120/ad	Ib	C	*cat*
220, 339	Group-housed cats	Typhimurium	120/ad	Ic	C	*cat*

^a^A, ampicillin; C, chloramphenicol; Su, sulfonamides, T, tetracycline; Sxt, sulfamethoxazole-trimethoprim; ND, not determined; PFGE, pulsed-field gel electrophoresis. ^b^Roman numerals indicate the major types of fragment patterns; lowercase letters indicate minor variations in the respective fragment pattern.

### Invasion of the Human Intestinal Epithelial Cell Line T84

**Figure 2:**
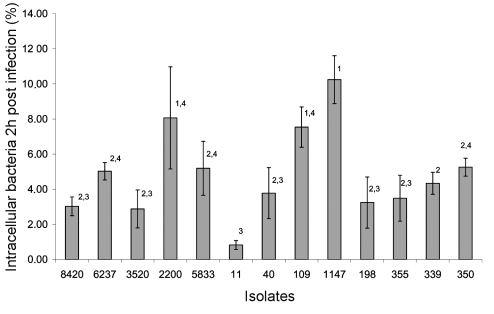
Invasion of *Salmonella* strains in the human intestinal epithelial cell line T84. The y-axis shows the percentages of intracellular bacteria 2 hours postinfection, relative to the initial number of bacteria, incubated with the cells. The x-axis shows isolate numbers. All isolates derived from the group-housed cats had the same invasion percentage as strains 198 and 355 (data not shown). Isolates 55 and 1145 had the same invasion percentage as strain 1147 (data not shown). Data not sharing superscript numbers indicate statistically significant differences (p < 0.05).

All isolates invaded T84 cells, with the cat isolates of *S*. Typhimurium PT193 (strains 1147, 1145, and 55, which belong to the same clone) and the human isolate *S*. Typhimurium strain 2200, the most invasive, yielding a percentage of invasion of 8% to 10%. The multidrug-resistant cat isolate *Salmonella* 4:i:- (strain 11) was the least invasive strain, having an invasion percentage of about 0.5%. Invasion percentages of the different isolates are shown in Figure 2. Of the strains of the same PFGE type, only one was shown in Figure 2, since no significant differences were detected between the invasion percentages of these strains. Statistically significant differences are shown in the figure.

## Discussion

This study concluded that, although cats can transmit *Salmonella* strains, healthy house cats are generally safe. Earlier reports regarding isolation of pathogens from healthy cats showed low percentages (mostly around 1%) of *Salmonella*-positive rectal swabs (*18**,**19*). In our study, 1 of 278 healthy cats was found to be positive. Immunodeficiency and nonhygienic housing can be predisposing factors for cats to shed *Salmonella* in the feces, resulting in contamination of the environment. Rectal swabs from 18 of 35 group-housed kittens were *Salmonella*-positive in our study. The fact that the 35 kittens were derived from more than 10 different owners before being group-housed and that one PFGE type (three subtypes) of *S*. Typhimurium 120/ad was isolated, indicates spread of the *Salmonella* strain between the cats or a common source. The age of these animals may also play a role, since all animals in this group were <4 months. Young animals are more susceptible to *Salmonella* infection. Also immunodeficiency can result in *Salmonella* excretion. One outbreak of fatal salmonellosis in cats has been reported after mild immunosuppression induced by live panleukopenia virus vaccination (*7*). In our study, animals infected with FIV and one animal that had panleukopenia shed *Salmonella*. Three animals that were infected with FIV were derived from the same owner, which indicates that the animals were infected with *Salmonella* from the same source or that one animal contaminated the others.

In our study, serotypes Typhimurium, Enteritidis, Bovismorbificans, and 4:i:- were isolated from cats. The isolated serotypes indicate that the cats were infected from the same sources compared with other animals and man. Indeed, serotypes Typhimurium and Enteritidis are the most widespread serotypes and the serotype Bovismorbificans is not uncommon in other animals, including humans (*2**,**20*).

Generally, invasion in the human intestinal epithelial cell line T84 was comparable between the cat isolates and isolates from humans. Invasion in intestinal epithelial cells is the primary step in the pathogenesis of *Salmonella* that causes gastrointestinal problems (*21*). This finding implies that the cat isolates are potentially pathogenic for humans. Moreover, all cat isolates harbored the *SopB* gene, which is involved in blocking the closure of chloride channels in gut epithelium and thus in inducing diarrhea. As in most other animal species, the cat isolates of the serotype Typhimurium harbored antimicrobial drug–resistant genes, raising concerns about spreading antimicrobial drug–resistant strains to humans.

Since the 1990s, concerns have arisen about the emergence and spread of multidrug-resistant Typhimurium strains, especially the multidrug-resistant ACSSuT type, which is resistant to ampicillin, chloramphenicol, streptomycin, sulfonamides, and tetracycline (*2*). In our study, some *S*. Typhimurium isolates from cats were resistant to a single drug such as ampicillin or chloramphenicol, while most isolates from the group-housed cats (same clone) were resistant to ampicillin, chloramphenicol, and tetracycline. Resistance genes were found to be *bla_TEM_* (ampicillin), *cat* (chloramphenicol), and *tet*(*A*) (tetracycline). The genes in the class 1 integron of the multidrug-resistant genomic island in ACSSuT type *S*. Typhimurium, required for the resistances to the above three mentioned antimicrobial drugs, are *bla_PSE1_*, *floR*, and *tet*(*G*) (*22*). This illustrates that these isolates did not acquire their resistance genes from horizontal transfer from pentadrug-resistant ACSSuT type strains. The isolate *Salmonella* 4:i:- was resistant to ampicillin, chloramphenicol, sulfonamides, tetracycline, and sulfamethoxazole/trimethoprim (ACSuTSxt-type), encoded by *bla_TEM_* (ampicillin), *cat* (chloramphenicol), *sul2* (sulfonamides), *tet*(*A*) (tetracycline), and *dfrA1* (trimethoprim). Also the resistance shown by this example had no relationship to the typical *S*. Typhimurium DT104 multidrug-resistant genomic island.

In conclusion, healthy house cats are generally safe with regard to excretion of *Salmonella* in the environment. Cats that are sick or are receiving medication resulting in immune deficiencies can potentially pose a threat to public health. Young children, the elderly, and immunocompromised persons are at risk because of their high sensitivity for the infection. All persons should follow good hygiene practices when keeping cats as pets.
